# Supplementary material for: Characterization of Yeasts Isolated from Parmigiano Reggiano Cheese Natural Whey Starter: From Spoilage Agents to Potential Cell Factories for Whey Valorization
Source: Microorganisms. 2021 Nov 3;9(11):2288. doi: 10.3390/microorganisms9112288 (PMC8623691; doi:10.3390/microorganisms9112288)
Supplement: Supplementary file 1 [file microorganisms-09-02288-s001.zip › microorganisms-1396526-supplementary.pdf]

microorganisms

MDPI

Article

Characterization of Yeasts Isolated from Parmigiano Reggiano Cheese Natural Whey Starter: From Spoilage Agents to Potential Cell Factories for Whey Valorization

Serena Martini [1], Mattia Bonazzi [1], Ilaria Malorgio [1], Valentina Pizzamiglio [2], Davide Tagliazucchi [1], Lisa Solieri [12,*]

[1] Department of Life Sciences, University of Modena and Reggio Emilia, Via Amendola, 2 - Pad. Besta, 42122 Reggio Emilia, Italy; serena.martini@unimore.it (S.M.); 240824@studenti.unimore.it (M.B.); malorgioilaria.94@gmail.com (I.M.); davide.tagliazucchi@unimore.it (D.T.)

[2] Consorzio del Formaggio Parmigiano Reggiano, via J.F. Kennedy 18, 42124, Reggio Emilia, Italy; pizzamiglio@parmigianoreggiano.it

* Correspondence: lisa.solieri@unimore.it; Tel.: +39 0522 522026

Abstract: Whey is the main by-product of the dairy industry and contains sugars (lactose) and proteins (especially serum proteins and, at lesser extent, residual caseins), which can be valorized by the fermentative action of yeasts. In the present study, we characterized the spoilage yeast population inhabiting natural whey starter (NWS), the undefined starter culture of thermophilic lactic acid bacteria used in Parmigiano Reggiano (PR) cheesemaking, and evaluated thermotolerance, mating type, and the aptitude to produce ethanol and bioactive peptides from whey lactose and proteins, respectively, in a selected pool of strains. PCR-RFLP assay of ribosomal ITS regions and phylogenetic analysis of 26S rDNA D1/D2 domains showed that PR NWS yeast population consists of the well-documented *Kluyveromyces marxianus*, as well as of other species

(*Saccharomyces cerevisiae*, *Wickerhamiella pararugosa*, and *Torulaspora delbrueckii*), with multiple biotypes scored within each species as demonstrated by $(GTG)_5$-based MSP-PCR. Haploid and diploid *K. marxianus* strains were identified through *MAT* genotyping, while thermotolerance assay allowed the selection of strains suitable to grow up to 48 °C. In whey fermentation trials, one thermotolerant strain was suitable to release ethanol with a fermentation efficiency of 86.5%, while another candidate was able to produce the highest amounts of both ethanol and bioactive peptides with potentially anti-hypertensive function. The present work demonstrated that PR NWS is a reservoir of ethanol and bioactive peptides producer yeasts, which can be exploited to valorize whey, in agreement with the principles of circularity and sustainability.

Keywords: Parmigiano Reggiano cheese; whey; natural whey starter; ethanol; bioactive peptide; yeasts; *Kluyveromyces marxianus*; *Wickerhamiella pararugosa*; *Torulaspora delbrueckii*

Citation: Martini, S.; Bonazzi, M.; Malorgio, I.; Pizzamiglio, V.; Tagliazucchi, D.; Solieri, L. Characterization of Yeasts Isolated from Parmigiano Reggiano Cheese Natural Whey Starter: From Spoilage Agents to Potential Cell Factories for Whey Valorization. *Microorganisms* **2021**, *9*, 2288. https://doi.org/10.3390/microorganisms9112288

Academic Editor: Salam A. Ibrahim

Received: 10 September 2021
Accepted: 29 October 2021
Published: 3 November 2021

Publisher's Note: MDPI stays neutral with regard to jurisdictional claims in published maps and institutional affiliations.

Copyright: © 2021 by the authors. Licensee MDPI, Basel, Switzerland. This article is an open access article distributed under the terms and conditions of the Creative Commons Attribution (CC BY) license (https://creativecommons.org/licenses/by/4.0/).

Supplementary Data

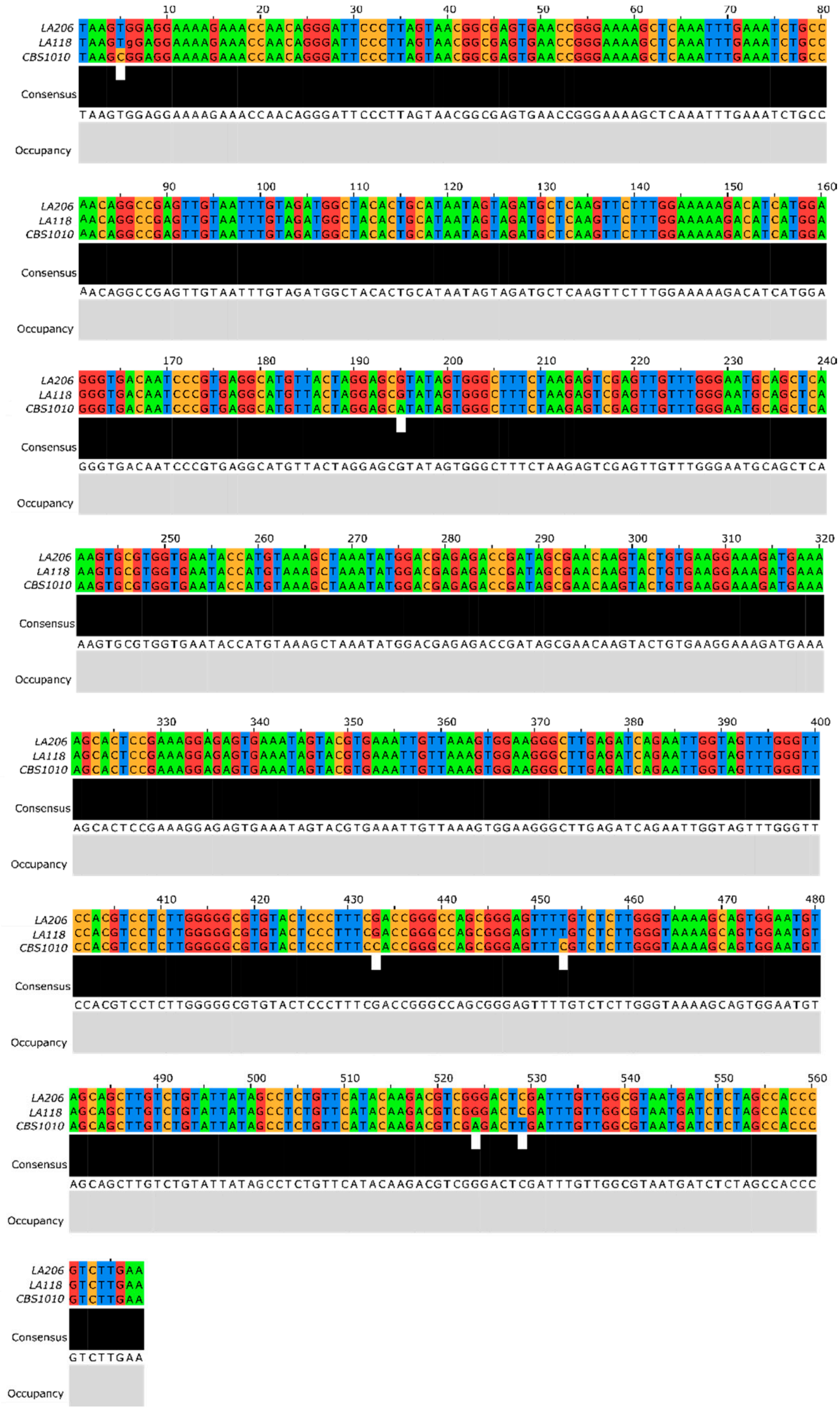

Figure S1. Multiple sequence alignment of 26S rRNA D1/D2 domain nucleotide sequences of *W. pararugosa* LA118 and LA206 with reference strain CBS1010^{T}. Nucleotide sequences were aligned using Muscle program [39] in MEGA X software [40] and the resulting alignment was visualized using JalView v2.11 [64].

Table S1. Kinetic growth parameters of selected 12 *K. marxianus* strains, 2 *W. pararugosa* and 1 *T. delbrueckii* strain. Growth data obtained by gravimetric assays in whey were fitted with different mathematical models in Grofit [50] to calculate growth rate μ (h^{-1}), growth efficiency A (maximum % of CO_2 released), and lag phase (h). When available, for each strains *MAT* genotype and temperature score are reported. Data are represented as mean (n=3) ± standard deviation. Abbreviations: T, temperature; Km, *K. marxianus*; Wp, *W. pararugosa*; Td, *T. delbrueckii*; nd, not detected.

				Growth parameters			
**Species**	**Strains**	**Mating type**	**T score**	**Model**	**μ**	**A**	**λ**
Km	CA104	*MATa/MATα*	13	Richards	0.0206 ±0.0009	2.3614±0.0460	25.1589±1.7176
	CA105	*MATα*	13	Richards	0.0192±0.0009	2.1657±0.3203	67.7658±3.6328
	CA111	*MATa/MATα*	13	Gompertz	0.0233±0.0047	2.6124±0.2264	15.10322.3275
	CA116	*MATa/MATα*	15	Richards	0.0170±0.0002	1.9749±0.6838	69.2531±7.3062
	CA204	*MATa/MATα*	16	Richards	0.0197±0.0009	2.0128±0.4480	31.8511±2.2288
	CA207	*MATa/MATα*	9	logistics	0.0230±0.0004	1.7240±0.1685	45.4162±10.7960
	CA213	*MATa/MATα*	12	Richards	0.0241±0.0006	2.3682±0.3023	30.8106±9.0832
	CA214	*MATa/MATα*	12	logistics	0.0222±0.0018	2.0853±0.4113	35.1206±12.0657
	LA102	*MATα*	12	Gompertz	0.0241±0.0011	2.2359±0.3692	19.0651±2.5031
	LA202	*MATα*	15	logistics	0.0219±0.0019	2.2831±0.0103	78.8519±11.8921
	LA210	*MATα*	12	Gompertz	0.0218±0.0008	2.5022±0.3536	21.2292±6.1693
	RO101	*MATa*	12	Richards	0.0215±0.0022	2.0656±0.2168	43.9611±1.8880
Wp	LA118	nd	nd	Gompertz	0.0077±0.0000	0.1493±0.0000	3.434038±0.0000
	LA218	nd	nd	modified Gompertz	0.007±0.00004	0.4814±0.0000	10.67506±0.0000
Td	RO204-3	nd	nd	Gompertz	0.0040±0.0021	2.8294±0.3888	326.5984±10.8513

Table S2. β-casein-derived peptides identified after whey fermentation by selected yeast strains. Details on strain isolation are in Table 2.

Peptide sequence	Fragment	*m/z* observed	*m/z* expected	ppm	Charge	CA105	CA116	CA214	LA202	RO101	RO204-3
PGEIVE	9-14	643.3300	643.3297	-0.5	1			X		X	
SLSssEESITR	15-25	678.2598	678.2600	0.3	2			X	X		
SLsssEESITR	15-25	718.2449	718.2432	-2.4	2	X	X	X	X	X	
sLsssEESITR	15-25	758.2262	758.2263	0.1	2			X	X	X	
SLSssEESITRINK	15-28	570.9159	570.9164	0.9	3			X		X	
SLsssEESITRINK	15-28	895.8542	895.8542	0.0	2 and 3	X	X	X		X	X
sLsssEESITRINK	15-28	935.8392	935.8373	-2.0	2	X	X	X	X	X	X
SLsssEESITRINKK	15-29	640.2699	640.2702	0.5	3					X	
sLsssEESITRINKK	15-29	999.8848	999.8848	0.0	2						X
sssEESITRINK	17-28	795.7975	795.7961	-1.8	2					X	
KKIEKFQsE	28-36	608.8021	608.8023	0.3	2		X			X	
KIEKFQsEE	29-37	609.2757	609.2761	0.7	2					X	
DELQ	43-46	504.2277	504.2300	4.6	1					X	
ELQ	44-46	389.2014	389.2031	4.4	1					X	
AQTQSLVYPFPGPIPN	53-68	864.9530	864.9514	-1.8	1	X					
SLVYPFPGP	57-65	976.5151	976.5138	-1.3	1 and 2			X		X	
SLVYPFPGPI	57-66	545.3047	545.3026	-3.9	2	X					
SLVYPFPGPIPN	57-68	1300.6944	1300.6936	-0.6	1	X					
VYPFPGPIPN	59-68	1100.5787	1100.5775	-1.1	1	X	X		X	X	
SLPQ	69-72	444.2446	444.2453	1.6	1			X			
NIPPLTQTPVVVPP	73-86	736.4298	736.4296	-0.3	2					X	
NIPPLTQTPVVVPPF	73-87	809.9645	809.9638	-0.9	2	X					
IPPL	74-77	439.2925	439.2915	-2.3	1	X					
PPL	75-77	326.2076	326.2074	-0.6	1		X				
PPLTQTPV	75-82	852.4809	852.4825	1.9	1				X		
PPLTQTPVVVPP	75-86	622.8670	622.8661	-1.4	2			X		X	
PPLTQTPVVVPPF	75-87	696.4004	696.4003	-0.1	2	X					
PVVVPP	81-86	607.3817	607.3814	-0.5	1	X	X	X		X	

PVVVPPF	81-87	754.4492	754.4498	0.8	1 and 2	X	X	X	X	X	X
PVVVPPFLQP	81-90	1092.6431	1092.6452	1.9	1 and 2	X					
VVPP	83-86	411.2597	411.2602	1.2	1			X			
VMGVSK	92-97	310.6752	310.6754	0.6	2			X		X	X
VSKVKEA	95-101	380.7317	380.7318	0.3	2				X		
EAMAPK	100-105	646.3225	646.3229	0.6	1			X			
AMAPK	101-105	517.2801	517.2803	0.4	1			X	X	X	
APKHKEMPFPKYPVEPFT	103-120	715.0393	715.0397	0.6	4 and 3						X
LTD	127-129	348.1761	348.1765	1.1	1	X		X		X	
TDV	128-130	334.1603	334.1609	1.8	1			X			
DVENLHLPLP	129-138	573.8141	573.8113	-4.9	2	X	X	X		X	
VENLHLPLP	130-138	516.2982	516.2978	-0.8	2	X	X	X		X	
NLHLPLPLLQS	132-142	622.8748	622.8717	-5.0	2	X					
LHLPLP	133-138	345.2207	345.2209	0.6	2	X	X	X	X	X	
LHLPLPLLQ	133-141	522.3356	522.3342	-2.7	2	X					
LHLPLPLLQS	133-142	565.8508	565.8502	-1.1	2	X					
MHQPHQPLPPT	144-154	641.8222	641.8217	-0.8	2 and 3	X	X	X	X	X	
MHQPHQPLPPTVMFPP	144-159	618.6448	618.6445	-0.5	3			X		X	
MHQPHQPLPPTVmFPP	144-159	623.9769	623.9761	-1.3	3		X				
HQPHQPLPPT	145-154	576.3020	576.3014	-1.0	2	X	X				
HQPHQPLPPTVMFPP	145-159	574.9644	574.9643	-0.2	3				X		
LPPT	151-154	427.2534	427.2551	4.0	1						X
TVm	154-156	366.1694	366.1693	-0.3	1	X					
SLSQSKVLPVP	164-174	577.8436	577.8426	-1.7	2			X		X	
SLSQSKVLPVPQ	164-175	641.8722	641.8719	-0.5	2	X	X		X	X	X
SLSQSKVLPVPQKAVPYPQ	164-182	689.3940	689.3930	-1.5	3		X				X
SLSQSKVLPVPQKAVPYPQR	164-183	556.3224	556.3218	-1.1	4	X	X		X		X
SLSQSKVLPVPQKAVPYPQRDMPI	164-187	670.3743	670.3729	-2.1	4		X				
SLSQSKVLPVPQKAVPYPQRDMPIQ	164-188	936.1814	936.1810	-0.4	3						X
SQSKVLPVP	166-174	954.5622	954.5619	-0.3	1			X		X	
SQSKVLPVPQ	166-175	541.8137	541.8139	0.4	2	X	X	X	X	X	X

SQSKVLPVPQK	166-176	605.8611	605.8613	0.3	2	X		X	X	X	
SQSKVLPVPQKAVPYPQ	166-182	622.6869	622.6876	1.1	2 and 3						X
SQSKVLPVPQKAVPYPQR	166-183	506.2928	506.2928	0.0	3 and 4	X	X		X		
SQSKVLPVPQKAVPYPQRDMP	166-186	592.0749	592.0729	-3.4	4					X	
SKVLPVP	168-174	739.4700	739.4713	1.8	1			X			
SKVLPVPQ	168-175	867.5309	867.5298	-1.3	1			X		X	
SKVLPVPQK	168-176	498.3161	498.3160	-0.2	2					X	
KVLPVP	169-174	652.4377	652.4392	2.3	1 and 2			X		X	
KVLPVPQ	169-175	780.4978	780.4978	0.0	1	X		X		X	
KAVPYP	176-181	674.3879	674.3872	-1.0	1	X					
KAVPYPQ	176-182	802.4453	802.4458	0.6	1	X	X	X	X	X	
KAVPYPQR	176-183	479.7782	479.7771	-2.3	2	X	X	X	X	X	
LYQEPVLGPVRGPFP	192-206	834.9592	834.9590	-0.2	2					X	
YQEPVLGPVR	193-202	579.3149	579.3139	-1.7	2	X		X	X	X	
YQEPVLGPVRGPF	193-205	486.9296	486.9295	-0.2	2	X		X		X	
YQEPVLGPVRGPFP	193-206	778.4170	778.4170	0.0	2	X	X		X	X	
GPVRGPFP	199-206	413.7315	413.7321	1.5	2	X	X	X	X	X	
GPVRGPFPI	199-207	470.2746	470.2742	-0.9	2		X			X	
GPVRGPFPII	199-208	526.8174	526.8162	-2.3	2					X	X
IIV	207-209	344.2546	344.2544	-0.6	1	X	X	X	X	X	

Table S3. *α*S1-casein-derived peptides identified after whey fermentation by selected yeast strains. Details on strain isolation are in Table 2.

Peptide sequence	Fragment	*m/z* observed	*m/z* expected	ppm	Charge	CA105	CA116	CA214	LA202	RO101	RO204-3
RPKHPIKH	1-8	338.2100	338.2103	0.9	3			X			
RPKHPIKHQ	1-9	380.8963	380.8965	0.5	3	X		X	X		X
RPKHPIKHQGLPQEV	1-15	441.7562	441.7561	-0.2	4		X				
RPKHPIKHQGLPQEVL	1-16	470.0269	470.0272	0.6	4 and 5						X
LLR	20-22	401.2866	401.2871	1.2	1			X	X	X	
LSKDIGsEsTEDQ	40-52	784.7913	784.7919	0.8	2					X	
SKDIGsEsTE	41-50	606.7067	606.7071	0.7	2			X	X		
sKDIGsEsTE	41-50	646.6900	646.6902	0.3	2				X		
SKDIGsEsTED	41-51	664.2193	664.2206	2.0	2			X		X	
SKDIGsEStED	41-51	664.2195	664.2206	1.7	2						
SKDIGsEsTEDQ	41-52	728.2496	728.2498	0.3	2	X	X	X	X	X	
sKDIGsEsTEDQ	41-52	768.2322	768.2330	1.0	2						
SKDIGsEsTEDQA	41-53	763.7695	763.7684	-1.4	2	X	X	X	X	X	
IGsEsTEDQ	44-52	563.1722	563.1729	1.2	2				X		
DQAMEDIKQ	51-59	539.2471	539.2477	1.1	2					X	
DIK	56-58	375.2233	375.2238	1.3	1					X	X
sVEQKHIQ	75-82	524.7439	524.7448	1.7	2	X		X		X	
sVEQKHIQK	75-83	588.7920	588.7923	0.5	2		X				
HIQ	80-82	397.2186	397.2194	2.0	1			X		X	X
LRLKKYKVPQ	99-108	424.9426	424.9432	1.4	3	X	X		X		X
LRLKKYKVPQL	99-109	347.2304	347.2302	-0.6	4		X		X		X
LRLKKYKVPQLEIVPN	99-114	646.7342	646.7348	0.9	3 and 4		X		X		X
LRLKKYKVPQLEIVPNsAEERLH	99-122	568.9167	568.9167	0.0	5						X
LRLKKYKVPQLEIVPNsAEERLHSMKEGIHAQ	99-130	637.8435	637.8435	0.0	6						X
LRLKKYKVPQLEIVPNsAEERLHSMKEGIHAQQKEPMIGVNQEL	99-142	742.1083	742.1086	0.4	7						X

RLKKYKVPQ	100-108	387.2484	387.2485	0.3	2 and 3	X	X		X	X	
RLKKYKVPQL	100-109	318.9603	318.9592	-3.4	4				X		
KKYKVPQ	102-108	445.7763	445.7765	0.4	2	X	X	X		X	
KKYKVPQLEIVPN	102-114	389.7360	389.7356	-1.0	4						X
LEIVPN	109-114	684.3916	684.3927	1.6	1				X	X	X
KEGIHAQQK	124-132	519.7874	519.7882	1.5	2	X	X			X	
sAEER	115-119	336.1229	336.1234	1.5	2			X			
PMI	134-136	360.1935	360.1952	4.7	1						X
PEL	147-149	358.1959	358.1973	3.9	1						X
YYV	165-167	444.2135	444.2129	-1.4	1			X	X		
APSFSDIPNPIGSEN	176-190	772.8647	772.8650	0.4	2			X			
APSFSDIPNPIGSENSEK	176-193	944.9503	944.9498	-0.5	2			X		X	
SFSDIPNPIGSENSE	178-192	796.8569	796.8574	0.6	2			X			
FSDIPNPIG	179-187	480.2458	480.2453	-1.0	2	X		X			X
FSDIPNPIGSE	179-189	588.2843	588.2826	-2.9	2		X		X		
FSDIPNPIGSENSE	179-192	753.3406	753.3414	1.1	2	X	X		X	X	X
FSDIPNPIGSENSEK	179-193	817.3906	817.3888	-2.2	2		X				X
SDIPNPIGSENSE	180-192	679.8064	679.8072	1.2	2				X		
SDIPNPIGSENSEK	180-193	743.8548	743.8546	-0.3	2						X
IPNPIGSEN	182-190	470.7412	470.7404	-1.7	2	X					
IPNPIGSENSE	182-192	578.7783	578.7777	-1.0	2	X			X		

Table S4. αS2-casein-derived peptides identified after whey fermentation by selected yeast strains. Details on strain isolation are in Table 2.

Peptide sequence	Fragment	*m/z* observed	*m/z* expected	ppm	Charge	CA105	CA116	CA214	LA202	RO101	RO204-3
SIIsQETYK	13-21	574.7645	574.7654	1.6	2			X			
AINPsKENL	27-35	533.2531	533.2524	-1.3	2			X			
YSI	52-54	382.1975	382.1973	-0.5	1						X
YLYQGPIVLNPWDQVKR	98-114	697.0474	697.0458	-2.3	3		X	X		X	
LYQGPIVLNPWDQVKR	99-114	963.5336	963.5334	-0.2	2. 3 and 4	X	X	X	X	X	X
YQGPIVLNPWDQVK	100-113	828.9404	828.9408	0.5	2	X				X	
YQGPIVLNPWDQVKR	100-114	906.9917	906.9914	-0.3	2 and 3	X	X	X	X	X	X
QGPIVLNPWDQVK	101-113	747.4103	747.4092	-1.5	2						
QGPIVLNPWDQVKR	101-114	550.6424	550.6423	-0.2	3			X	X		
RNAVPITPT	114-122	484.7813	484.7798	-3.1	2		X				
NAVPITPTLNR	115-125	598.3433	598.3433	0.0	2	X	X	X	X	X	
NAVPITPTLNRE	115-126	662.8637	662.8646	1.4	2	X	X	X	X	X	
NAVPITPtLNREQLSTsEENSKKTVDMEsTEVF TKKTKLTEEEKNRLNF	115-163	842.9743	842.9755	1.4	7						
AVPITPTLNR	116-125	541.3234	541.3218	-3.0	2	X		X	X	X	
AVPITPTLNRE	116-126	605.8429	605.8431	0.3	2			X			
PITP	118-121	427.2534	427.2551	4.0	1						X
NREQLsTsEE	124-133	676.7419	676.7420	0.1	2			X			
YLK	179-181	423.2607	423.2602	-1.2	1	X	X	X		X	X
TKVIPYVR	198-205	325.8712	325.8710	-0.6	3	X	X				
YVRY	203-206	600.3153	600.3140	-2.2	1				X		

Table S5. κ-casein-derived peptides identified after whey fermentation by selected yeast strains. Details on strain isolation are in Table 2.

Peptide sequence	Fragment	*m*/*z* observed	*m*/*z* expected	ppm	Charge	CA105	CA116	CA214	LA202	RO101	RO204-3
KYIPIQYVL	30-38	568.8385	568.8393	1.4	2						X
YGL	44-46	718.2449	718.2432	-2.4	2	X				X	
PVALINN	53-59	740.4292	740.4301	1.2	1			X		X	
FLPYPYYAKPA	61-71	665.3473	665.3475	0.3	2					X	
AVRSPA	72-77	300.6766	300.6768	0.7	2			X		X	
AVRSPAQ	72-78	364.7059	364.7061	0.5	2			X			
AVRSPAQI	72-79	421.2485	421.2482	-0.7	2	X		X	X	X	
AVRSPAQIL	72-80	477.7898	477.7902	0.8	2				X		
SNTVPAK	86-92	358.7003	358.7005	0.6	2			X			
MAIPPKKNQ	112-120	342.8637	342.8637	0.0	2 and 3	X	X	X	X	X	X
MAIPPKKNQD	112-121	571.3056	571.3053	-0.5	2 and 3	X	X	X	X	X	X
MAIPPKKNQDK	112-122	635.3530	635.3528	-0.3	2 and 3	X	X	X	X	X	X
AIPPKKNQDK	113-122	569.8315	569.8326	1.9	2		X			X	
PPKKNQDK	115-122	477.7714	477.7720	1.3	2	X	X	X		X	
DKTEIPTINT	121-130	566.2986	566.2982	-0.7	2	X		X		X	X
KTEIPTIN	122-129	915.5132	915.5146	1.5	1			X		X	
TEI	123-125	362.1917	362.1922	1.4	1			X			
TEIPTIN	123-129	787.4177	787.4196	2.4	1	X				X	
TIASGE	130-135	577.2818	577.2828	1.7	1			X			
TIAsGEPT	130-137	855.3493	855.3496	0.4	1	X	X		X	X	X
TIAsGEPTSTPT	130-141	1241.5314	1241.5297	-1.4	1		X		X	X	
TIASGEPTSTPTTE	130-143	1391.6528	1391.6536	0.6	1					X	
TIAsGEPTSTPTTE	130-143	736.3129	736.3136	1.0	2					X	
TIASGEPTSTPTTEAVESTVATLEDsPE	130-157	967.4340	967.4322	-1.9	3					X	
GEPTSTPTTE	134-143	1019.4517	1019.4528	1.1	1			X	X		
STPTTE	138-143	635.2872	635.2883	1.7	1				X	X	
AVESTVAtL	144-152	970.4490	970.4493	0.3	1					X	
AVESTVATLEDsP	144-156	699.8041	699.8054	1.9	2					X	

AVEStVATLEDSPE	144-157	1527.6467	1527.6462	-0.3	1					X	
AVESTVATLEDsPE	144-157	764.3280	764.3267	-1.7	2					X	
AVEStVATLEDsPE	144-157	804.3109	804.3099	-1.2	2					X	
VESTVAtL	145-152	450.2102	450.2097	-1.1	2			X		X	
VESTVATLEDsPE	145-157	728.8079	728.8082	0.4	2					X	
STVATLEDsP	147-156	550.2322	550.2314	-1.5	2					X	
STVATLEDsPE	147-157	614.7537	614.7527	-1.6	3	X		X	X	X	
STVAtLEDsPE	147-157	654.7380	654.7358	-3.4	2	X		X	X	X	
TVATLEDsPE	148-157	571.2373	571.2367	-1.1	2			X		X	
VATLEDsPE	149-157	1040.4160	1040.4184	2.3	1 and 2			X		X	
TLEDsPE	151-157	870.3108	870.3128	2.3	1			X		X	
sPEVIESPP	155-163	1034.4446	1034.4442	-0.4	1					X	
sPEVIESPPEI	155-165	638.7897	638.7891	-0.9	2					X	
VIESPPEIN	158-166	499.2644	499.2637	-1.4	2		X	X	X		
SPPEIN	161-166	328.6655	328.6661	1.8	2			X		X	
SPPEINT	161-167	757.3737	757.3727	-1.3	1	X		X	X		
PEI	163-175	358.1959	358.1973	3.9	1						X

Table S6. α-lactalbumin-derived peptides identified after whey fermentation by selected yeast strains. Details on strain isolation are in Table 2.

Peptide sequence	Fragment	*m/z* observed	*m/z* expected	ppm	Charge	CA105	CA116	CA214	LA202	RO101	RO204-3
LTK	7-9	361.2440	361.2445	1.4	1			X		X	
TKCEVFR	8-14	441.7285	441.7287	0.5	2				X		
RELKDLKGYGGVSLPE	14-29	587.6575	587.6562	-2.2	3 and 4		X				X
ELKDLKGYGG	15-24	540.2909	540.2902	-1.3	2					X	
ELKDLKGYGGVSLPE	15-29	802.9291	802.9301	1.2	2 and 3	X	X				
KDLKGY	17-22	362.2047	362.2054	1.9	2	X					
KDLKGYGGVS	17-26	512.2777	512.2771	-1.2	2	X	X	X	X	X	
KDLKGYGGVSL	17-27	379.5489	379.5485	-1.1	3	X					
KDLKGYGGVSLP	17-28	411.9002	411.8994	-1.9	2 and 3	X	X	X	X	X	
KDLKGYGGVSLPE	17-29	454.9143	454.9136	-1.5	2 and 3	X	X	X	X	X	X
DLK	18-20	375.2233	375.2238	1.3	1					X	X
DLKGYGG	18-24	355.1796	355.1794	-0.6	2	X			X	X	
DLKGYGGVSLP	18-28	553.3002	553.2980	-4.0	2	X	X	X	X	X	
DLKGYGGVSLPE	18-29	617.8192	617.8193	0.2	2	X	X	X	X	X	X
KGYGGVS	20-26	667.3404	667.3410	0.9	1	X		X		X	
KGYGGVSLPE	20-29	503.7634	503.7639	0.9	2	X	X		X		
FHTSGYDT	35-42	464.1959	464.1958	-0.2	2	X					
FHTSGYDTQ	35-43	528.2257	528.2251	-1.1	2		X	X	X	X	
IVQNNDSTEYGL	45-56	676.8218	676.8201	-2.5	2						X
YGL	54-56	352.1875	352.1867	-2.3	1	X				X	
LDD	85-87	362.1551	362.1558	1.9	1	X	X		X		X
LTD	89-91	348.1761	348.1765	1.1	1	X		X		X	
KKIL	97-100	501.3756	501.3759	0.6	1		X				
ILD	99-101	360.2131	360.2129	-0.6	1	X	X	X	X	X	X
LDK	100-102	375.2240	375.2238	-0.5	1	X			X	X	X
DKVGINY	101-107	808.4184	808.4199	1.9	1			X		X	
DKVGINYW	101-108	497.7530	497.7533	0.6	2				X		
KVGINYW	102-108	440.2388	440.2398	2.3	2			X	X		
VGINYWLAHKA	103-113	424.5687	424.5680	-1.6	3		X				

LAH	109-111	340.1972	340.1979	2.1	1			X	X		
KLDQ	118-121	503.2803	503.2824	4.2	1						X

Table S7. β-lactoglobulin-derived peptides identified after whey fermentation by selected yeast strains. Details on strain isolation are in Table 2.

Peptide sequence	Fragment	*m/z* observed	*m/z* expected	ppm	Charge	CA105	CA116	CA214	LA202	RO101	RO204-3
LIV	2-4	344.2546	344.2544	-0.6	1	X	X	X	X	X	
LIVTQ	2-6	573.3606	573.3606	0.0	1	X					
LIVTQTMK	2-9	933.5435	933.5438	0.3	1 and 2	X	X	X	X	X	X
LIVTQTMKG	2-10	495.7869	495.7863	-1.2	2	X	X	X			
LIVTQTMKGL	2-11	552.3286	552.3283	-0.5	2	X	X	X	X	X	
LIVTQTMKGLD	2-12	609.8426	609.8418	-1.3	2	X		X	X	X	
LIVTQTMKGLDIQ	2-14	730.4143	730.4131	-1.6	2		X			X	
LIVTQTMKGLDIQK	2-15	794.4595	794.4606	1.4	2 and 3						X
LIVTQTmKGLDIQKVAGTW	2-20	706.7270	706.7258	-1.7	3	X	X				X
KGL	9-11	317.2185	317.2183	-0.6	1	X	X	X	X	X	
KGLDIQKVAGTW	9-20	439.2506	439.2505	-0.2	3		X				
LDI	11-13	360.2128	360.2129	0.3	1					X	
YSL	21-23	382.1975	382.1973	-0.5	1						X
AMAASDISL	24-32	439.7176	439.7180	0.9	2						X
AmAASDISLLDAQSAPLRVY	24-43	703.3625	703.3613	-1.7	3						X
SDISLLDAQSAPLRV	28-42	792.9347	792.9332	-1.9	2						X
SDISLLDAQSAPLRVY	28-43	874.4660	874.4649	-1.3	2 and 3						X
ISLL	30-33	445.3024	445.3021	-0.7	1					X	
ISLLDAQSAPLRV	30-42	691.9055	691.9037	-2.6	2						X
ISLLDAQSAPLRVY	30-43	773.4354	773.4354	0.0	2						X
LLDAQSAPLRVY	32-43	673.3780	673.3774	-0.9	2						X
LDAQSAPLRVY	33-43	616.8360	616.8353	-1.1	2		X				X
LDAQSAPLRVYVE	33-45	730.8909	730.8908	-0.1	2						X
DAQSAPLRV	34-42	478.7625	478.7616	-1.9	2		X			X	X
DAQSAPLRVYVE	34-45	674.3496	674.3488	-1.2	2	X	X		X	X	
AQSAPLR	35-41	371.7136	371.7139	0.8	2	X					

AQSAPLRV	35-42	421.2496	421.2482	-3.3	2	X				X	
AQSAPLRVY	35-42	502.7804	502.7798	-1.2	2	X	X		X		X
AQSAPLRVYVE	35-44	616.8373	616.8353	-3.2	2	X	X	X	X	X	X
YVEELKPTPE	42-52	602.8094	602.8084	-1.7	2					X	
YVEELKPTPEGDLEIL	42-58	922.9819	922.9800	-2.1	2						X
VEELKPTPE	44-52	521.2761	521.2768	1.3	2	X	X		X		
VEELKPTPEGDLEIL	44-58	841.4501	841.4484	-2.0	2 and 3						X
KPT	47-49	345.2129	345.2132	0.9	1						
KKII	70-73	501.3756	501.3759	0.6	1		X				
IAE	73-75	332.1808	332.1816	2.4	1			X			
KTKIPAVFK	76-84	516.3357	516.3342	-2.9	2 and 3	X	X	X	X	X	X
KTKIPAVFKI	76-85	382.2538	382.2533	-1.3	3	X	X				
KTKIPAVFKID	76-86	420.5964	420.5956	-1.9	3	X	X	X	X	X	
AVF	81-83	336.1923	336.1918	-1.5	1		X				X
IDALNENK	85-92	458.7397	458.7404	1.5	2						
VLVL	93-96	443.3233	443.3228	-1.1	1			X	X	X	
VLVLDTDYK	93-101	533.2955	533.2950	-0.9	2			X		X	
LVR	123-125	387.2711	387.2714	0.8	1				X		
VDDEALEKF	129-137	533.2599	533.2586	-2.4	2	X		X			
EALEKFDKALKALPMHIRL	132-150	445.4613	445.4595	-4.0	5						X
DKALKALPMHIRL	138-150	377.2297	377.2301	1.1	4						X
ALKALP	140-145	306.7073	306.7076	1.0	2			X		X	
ALKALPMHIRL	140-150	316.4497	316.4496	-0.3	3 and 4	X	X	X	X	X	
KALPMHIR	142-149	322.5289	322.5286	-0.9	3	X					
KALPMHIRL	142-150	539.8311	539.8313	0.4	2 and 3	X	X	X	X	X	
PMHIRLS	145-151	427.2397	427.2393	-0.9	2	X		X			
HIRLSFNPT	147-155	542.7983	542.7985	0.4	2	X	X	X	X	X	
